# Physical properties of epilithic river biofilm as a new lead to perform pollution bioassessments in overseas territories

**DOI:** 10.1038/s41598-020-73948-7

**Published:** 2020-10-14

**Authors:** Dominique Monti, Cedric Hubas, Xavier Lourenço, Farid Begarin, Alexandre Haouisée, Laurence Romana, Estelle Lefrançois, Alexandra Jestin, Hélène Budzinski, Nathalie Tapie, Théo Risser, Jean-Louis Mansot, Philippe Keith, Olivier Gros, Pascal-Jean Lopez, Béatrice Lauga

**Affiliations:** 1 UMR BOREA, UA-MNHN-SU-IRD-CNRS-UCN, Université des Antilles, BP 592, 97157 Pointe-à-Pitre, Guadeloupe France; 2 Muséum National D’Histoire Naturelle, UMR BOREA, MNHN-SU-IRD-CNRS-UCN-UA, Place de la croix, Station Marine de Concarneau, Concarneau, France; 3 Muséum National D’Histoire Naturelle, UMR BOREA, MNHN-SU-IRD-CNRS-UCN-UA, 57 rue Cuvier, CP26, 75231 Paris Cedex 05, France; 4 C3MAG, UFR Des Sciences Exactes Et Naturelles, Université Des Antilles, BP 592, 97159 Pointe-à-Pitre, Guadeloupe France; 5 GTSI, département de Physique, Université des Antilles, BP 592, 97159 Pointe-à-Pitre Cedex, Guadeloupe France; 6 ECO in’EAU, 5 imp. Les Lambrusques, 34980 Montferrier-sur-Lez, France; 7 UPR 103 HORTSYS - CIRAD - Fonctionnement agroécologique Et Performances Des systèmes de Cultures Horticoles, Campus Agro-Environnemental Caraïbe, 97285 Le Lamentin, Martinique France; 8 UMR CNRS 5805 EPOC – OASU, Équipe LPTC, Université de Bordeaux, 351 Cours de la libération, 33405 Talence Cedex, France; 9 Institut de Systématique, Evolution, Biodiversité (ISYEB), Université Des Antilles, MNHN, CNRS, SU, EPHE, BP 592, 97157 Pointe-à-Pitre, Guadeloupe France; 10 E2S UPPA, CNRS, IPREM, Universite de Pau Et Des Pays de L’Adour, BP 1155, 64013 Pau Cedex, France

**Keywords:** Environmental impact, Tropical ecology

## Abstract

Chlordecone (CLD) levels measured in the rivers of the French West Indies were among the highest values detected worldwide in freshwater ecosystems, and its contamination is recognised as a severe health, environmental, agricultural, economic, and social issue. In these tropical volcanic islands, rivers show strong originalities as simplified food webs, or numerous amphidromous migrating species, making the bioindication of contaminations a difficult issue. The objective of this study was to search for biological responses to CLD pollution in a spatially fixed and long-lasting component of the rivers in the West Indies: the epilithic biofilm. Physical properties were investigated through complementary analyses: friction, viscosity as well as surface adhesion were analyzed and coupled with measures of biofilm carbon content and exopolymeric substance (EPS) production. Our results have pointed out a mesoscale chemical and physical reactivity of the biofilm that can be correlated with CLD contamination. We were able to demonstrate that epilithic biofilm physical properties can effectively be used to infer freshwater environmental quality of French Antilles rivers. The friction coefficient is reactive to contamination and well correlated to carbon content and EPS production. Monitoring biofilm physical properties could offer many advantages to potential users in terms of effectiveness and ease of use, rather than more complex or time-consuming analyses.

## Introduction

Out of the 11,435 French water bodies analysed for quality compliance according to regulations imposed by European legislation, 10% are located between the tropics and the equator, thousands of kilometres away from European shores^1^. In the outermost tropical territories, climatic characteristics often result in highly turbulent tropical streams^2^ that support simplified ecosystems with very few primary producers. Due to high river flow, perennial phytoplankton, zooplankton and macroalgae are scarce or missing and the epilithic biofilm deserves here, even more than elsewhere, the comparison with a real productive and contributive “microbial skin”^3^. The epilithic biofilm is the only endogenous long-lasting primary producer that grows on submersed river stones and is largely exploited as a food source by all diadromous fish or crustaceans^4,5^. In these countries, the simplified food webs and the massive flows of post-larvae and juveniles regularly re-entering the rivers and migrating upstream—typical of the diadromous life-cycle^6–8^—weaken the ability to correlate macrofaunal abundances with measures of environmental quality^9^.

Meanwhile, many rivers are contaminated by pesticides^10^ due to a tropical agronomy struggling with strong pest pressures. Chlordecone (CLD, Kepone®, Curlone®)^11^, the main pollutant of concern, was formerly used in banana tree plantations and remains strongly persistent. This molecule, banned from use in the 1990s, continues to be present in aquatic ecosystems and contaminates fish and crustaceans consumed by local populations with health-endangering consequences^12^. The effects of this molecule have been documented since the 1976 accident at the Hopewell Life Sciences Products Co. chemical factory, where the insecticide Kepone® was manufactured (Hopewell, Virginia, US) (for a detailed synopsis of the incident, see^13^). The maximum acceptable toxicant concentration in water was assessed to be between 1 and 2 μg/L for amphipods based on reproduction, growth, emergence, and survival^14^, making this pollutant an active molecule at a very low concentration. Previous research conducted in the Chesapeake Bay also showed significant impact of CLD on natural bacterial communities composition and growth at concentrations of 20 μg/L, both in the sediments and the water column^15–17^. In literature, bacteria have long been considered a precursor in the construction of epilithic biofilms, with the ability to facilitate the later establishment of more vulnerable and photosynthetic cells. The role of biotic interactions during very early biofilm is crucial for its construction^18^. One of the most important aspects of the relationship between bacteria and subsequent colonisers occurs through the production of EPS that not only plays a protective key role for bacteria^19,20^, but also in the attachment of colonies to the substrate^21–24^ with compounds that can either be activators or inhibitors of subsequent microalgal fixation^25,26^.

Among the methods used to study biofilms, hydraulic^27,28^ or mechanical properties have been a subject of interest since classical imaging techniques such as Confocal Laser Scanning Microscopy (CLSM) require the use of stains altering the structural and chemical properties of biofilms^29^. Most of this research pertains to conditions affecting the human body^30–32^ or biocorrosion processes^33,34^, but friction measurements in natural biofilms were also used to investigate its ecological significance^35^. Demonstrated positive correlation was established between friction and the size of bacterial microcolonies^30^ whose size and spatial distribution were shown strongly modulated by environmental conditions^36^.To qualify other physical properties, the Magnetic Particle Induction technique (MagPI), developed by Larson et al*.*^37^ provides precise determinations of biofilm adhesiveness at high temporal and mesoscale spatial resolution^38^ and has been applied successfully by several research groups working on aquatic biofilms^39–42^. Nanoindentation techniques, used in a dynamic mode, were successfully used to investigate the mechanical properties of the biofilms at a nanometric scale^43–45^ with a high spatial resolution^46^.

All these elements form the basis of the two hypotheses for this work: the epilithic biofilm, in its biological components, could serve as a bioindicator of CLD loads in Caribbean rivers and the biofilm’s general physical properties could reflect the modifications in contamination. In order to search for CLD bioindicators metrics in biofilms, we have integrated two levels of approaches: (a) an in situ study of diatomic assemblages in mature biofilms combined with chemical and physical analyses of the primo colonisation of artificial substrates located at stations different by their CLD loads and (b) a microcosm-based experiment to infer the effects of CLD contamination alone.

## Results

### Chlordecone quantification characterises three pollution contexts

**Figure 1:**
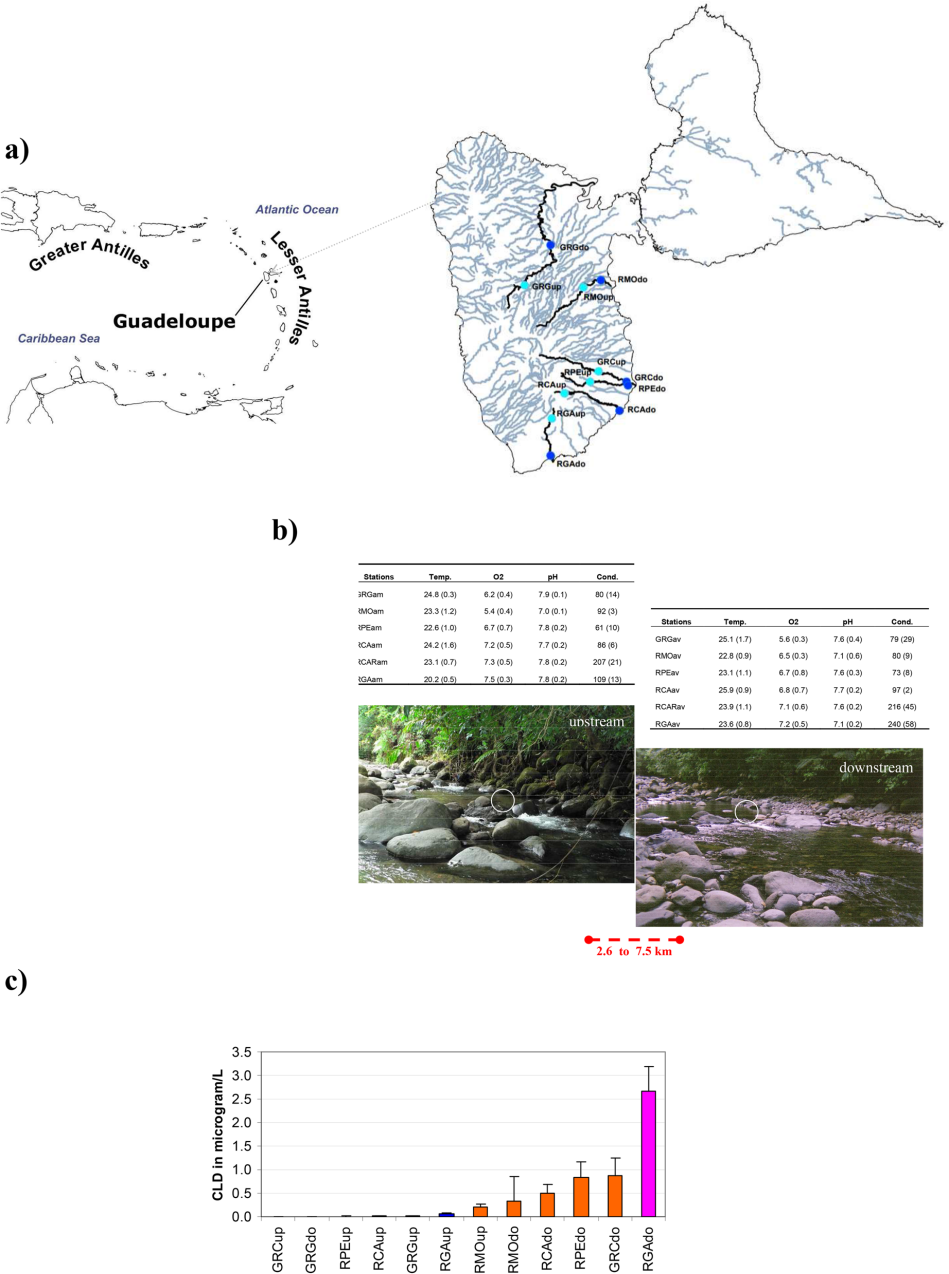
(**a**) Map of the sampling stations on the Basse-Terre region (French West Indies). Localisation of the upstream (lightblue) and downstream (darkblue) sampling sites in 6 different rivers. Grande rivière à Goyaves River: GRG, Moustique River: RMO, Grande rivière de Capesterre River: GRC, Pérou River: RPE, Carbet River: RCA, Grande Anse River: RGA. (**b**) Pictures showing the general configuration of the "upstream" and "downstream" stations, with values of temperature, O2 (mg/l), pH, conductivity, (s.d., N = 4) (**c**) Mean CLD contamination on the 12 stations.

In the rivers studied (Fig. 1a), the concentration of CLD in water ranged from undetectable to elevated (2.76 µg/L, see Table 1), with the highest values noted downstream of the Grande Anse River (RGAdo).

**Table 1:** Chlordecone (CLD) concentrations in the water at each station are based on four samples taken in February 2013. Downstream and upstream refer to the location of the sampling stations in relation to the banana plantations. T0 = slide immersion, T07, T14, T21 =  + 7, + 14, and + 21 days.

River	Location	Name of the station	CLD (µg/L)			
			T0	T07	T14	T21
Capesterre	Downstream	GRCdo	0.440	1.040	0.400	1.080
Carbet	Downstream	RCAdo	0.630	0.570	0.240	0.610
Grande Anse	Downstream	RGAdo	2.760	1.750	1.640	1.740
Grande Goyaves	Downstream	GRGdo	0.010	0.010	0.010	0.010
Moustique	Downstream	RMOdo	1.160	0.150	0.120	0.180
Pérou	Downstream	RPEdo	0.660	1.030	0.510	1.230
Capesterre	Upstream	GRCup	<	0.020	0.010	0.010
Carbet	Upstream	RCAup	0.001	<	<	<
Grande Anse	upstream	RGAup	0.120	0.040	0.040	0.090
Grande Goyaves	Upstream	GRGup	0.020	<	<	<
Moustique	Upstream	RMOup	0.240	0.250	0.110	0.210
Pérou	Upstream	RPEup	0.005	0.020	0.010	0.005

<, less than the analytical threshold.

All stations upstream from the banana plantations indicated a faint CLD pollution, except for the Moustique River (RMOup). In contrast, all stations downstream from the banana plantations were polluted by CLD, except for the Grande Goyaves River (GRGdo). Mean contamination values revealed three pollution contexts: a very limited or unpolluted context (between undetectable to 0.1 µg/L, Fig. 1b), a medium polluted context where values are compatible with those currently observed in the rivers of Guadeloupe and Martinique (from 0.1 to 1 µg/L), and one extreme situation at the RGA station (> 1 µg/L and up to 2.76 µg/L), downstream from the banana plantations.

### Epilithic diatom assemblages in mature biofilm did not reveal CLD contamination

**Figure 2:**
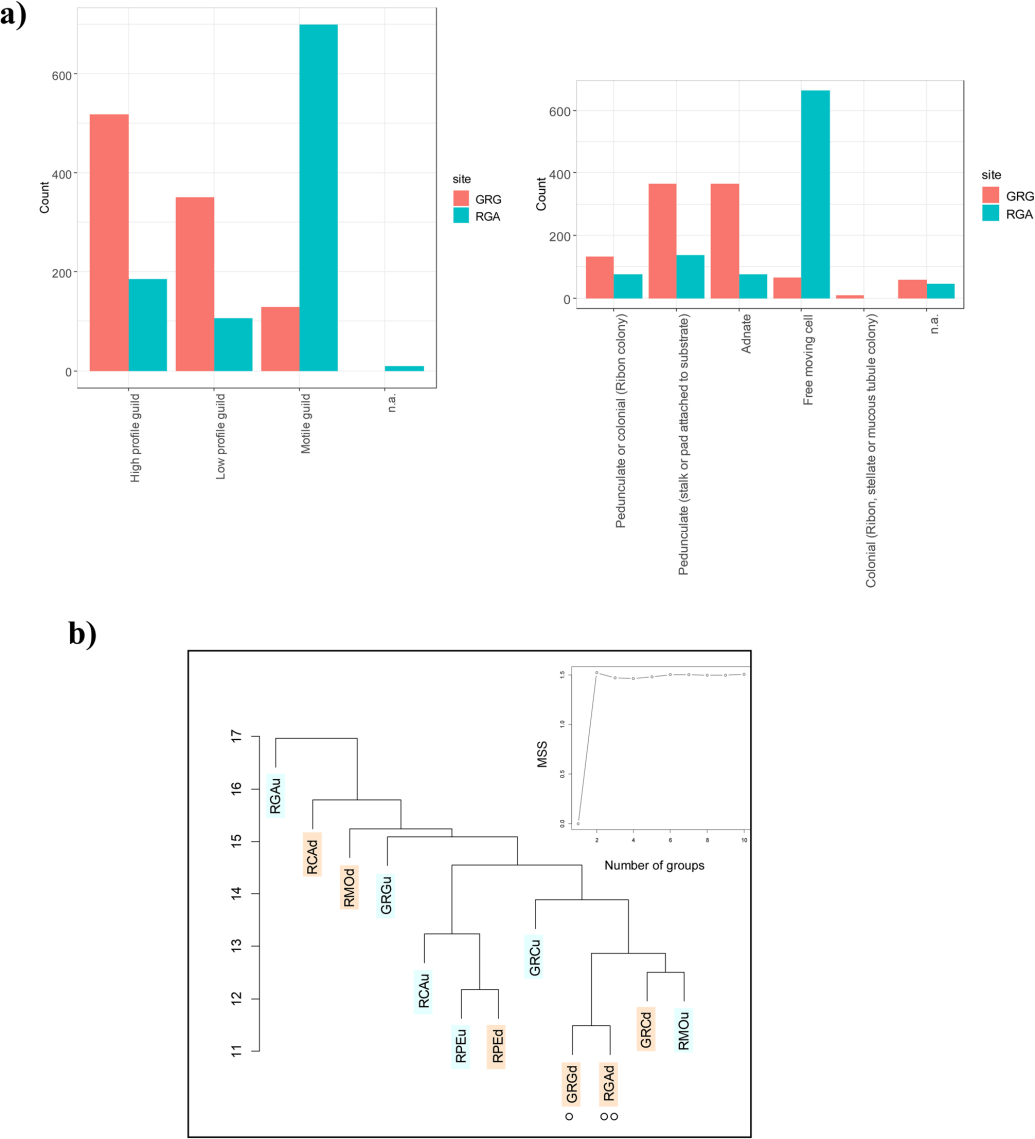
(**a**) Diatom species repartition in the two downstream stations (GRG and RGA) according to life form or guilds (relative abundance), (**b**) results of agglomerative hierarchical clustering of stations on the basis of their diatom assemblages, u: upstream (blue) and d: downstream (pink). The inset shows the Mean Split Silhouette (MSS) criterion for identification of optimal number of groups. ° and °° are respectively the less contaminated and the most contaminated stations. For abbreviations, see Table 1.

Ninety-nine epilithic diatom taxa were identified (Supp. dataset). Sixty-six percent of these were fully identified with a valid taxonomic appellation (those composed of a four-letter code), while other taxa were identified with a four-character code and a number. The specific richness was between 17 and 35 taxa per station, which is generally quite low, but common for the region. Only 2 to 8 species per station constitute each more than 5% of the abundance. All of the species are very common except one species inventoried at the RCA downstream station, which could be an indicator of organic or nutrient pollution: *Nitschia amphibia* (NAMP). Among the 23 dominant species, only 3 pioneer taxa belonging to the *Achnanthidium minutissimum* complex were inventoried. Three species were adnate (patched on substrate), 10 were pedunculate (pad or stalk to substrate) and 2 species were both pedunculate or able to form ribbon colonies: *Achnanthidium catenatum* and *Fragilaria fonticola*. None of the 23 dominant species were planktonic, 7 belong to the high-profile guild, 7 belong to the low profile guild and the other 9 belong to the motile guild. Diatoms inventoried at the GRGdo station were mainly attached to the substrate (adnate or pedunculate) and belong to the high or low profile guilds, while in contrast, the majority of diatoms at the RGAdo station were free moving cells (e.g., *Nitzschia inconspicua* [NINC], which was dominant) belonging to the motile guild (Fig. 2a). However, these two stations showed a proliferation of common species such as *Cocconeis euglypta* (CEUG) and *Gomphonema designatum* (GDES)*.*

Clustering based on diatomic assemblages revealed a loose tree structure, with scattered singletons (Fig. 2b). The two closest stations from a taxonomic perspective were paradoxically the two most distant stations from a contamination perspective: GRG as the least polluted, and RGA, the pollution of which represents a world record. The Mean Split Silhouette (MSS) did not reveal any improvement based on the number of groups, confirming the extreme heterogeneity of these assemblages. There was no visible aggregation between upstream or downstream stations or related to geographical proximity (such as stations located on the same river), except for the RPE river.

### Differences observed in EPS carbohydrates and proteins

**Figure 3:**
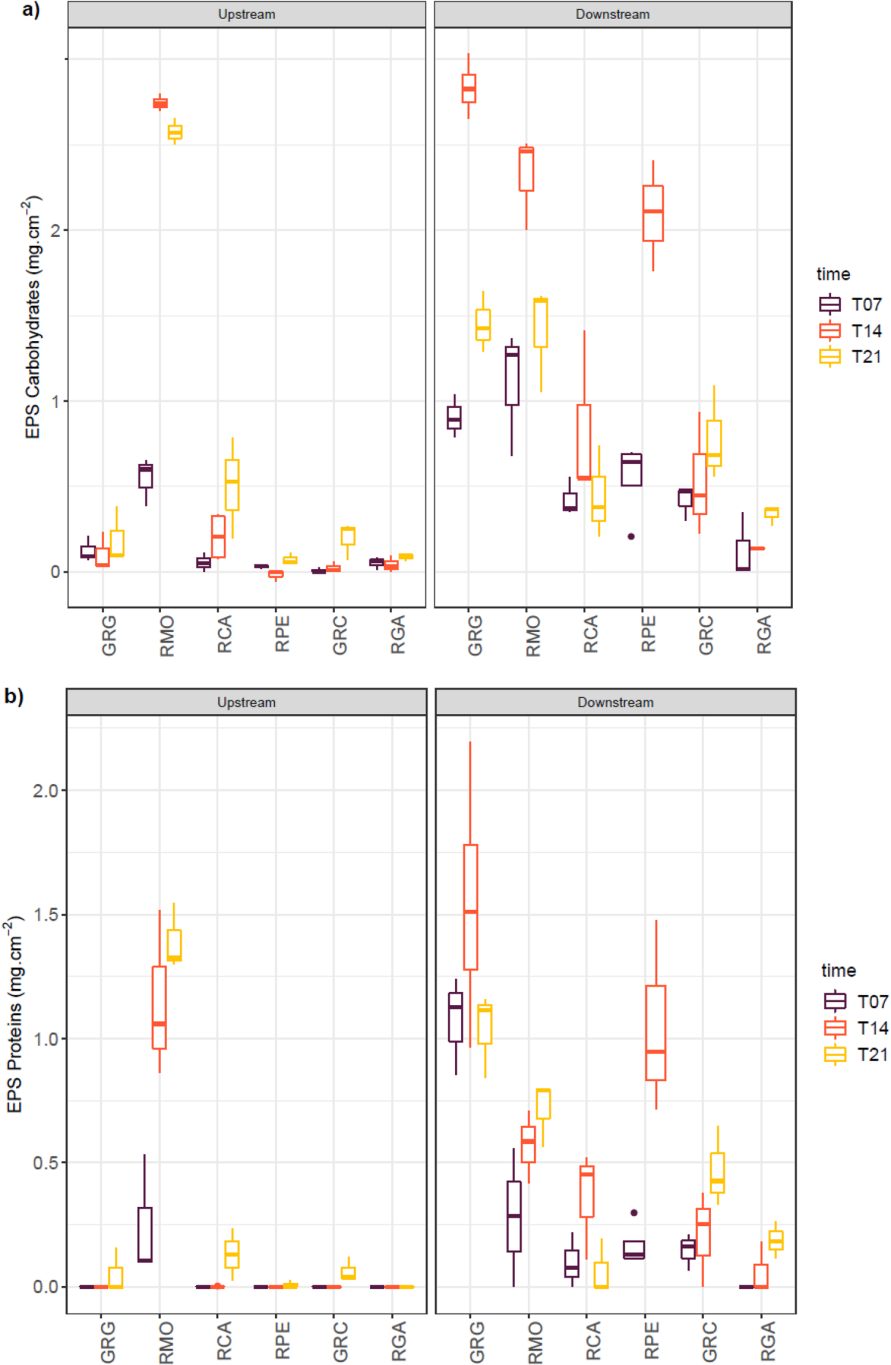
(**a**) Extracellular carbohydrates of upstream (left panel) and downstream (right panel) epilithic biofilms measured by colorimetry (mgEq Glucose/cm^2^). Sampling sites where ordered from the least polluted to the most polluted according to the order given in Fig. 1b for downstream sites, (**b**) extracellular proteins of upstream (left panel) and downstream (right panel) epilithic biofilms measured by colorimetry (mgEq BSA/cm^2^). Sampling sites were ordered from the least polluted to the most polluted according to the order given in Fig. 1b for downstream sites.

In both upstream and downstream sites, EPS measurements revealed differences in carbohydrate concentrations (Fig. 3a) (upstream: 2-way ANOVA; time effect F = 95.99, df = 2, *p* < 0.001; station effect F = 454.31, df = 5, *p* < 0.001; interaction F = 62.97, df = 10, *p* < 0.001; downstream: 2-way ANOVA; time effect F = 67.6, df = 2, *p* < 0.001; station effect F = 58.48, df = 5, *p* < 0.001; interaction F = 10.32, df = 9, *p* < 0.001) and in protein concentrations (Fig. 3b) based on the date and the station (upstream: 2-way ANOVA; time effect F = 21.43, df = 2, *p* < 0.001; station effect F = 107.93, df = 5, *p* < 0.001; interaction F = 14.95, df = 10, *p* < 0.001; downstream: 2-way ANOVA; time effect F = 14.68, df = 2, *p* < 0.001; station effect F = 33.65, df = 5, *p* < 0.001; interaction F = 2.99, df = 9, *p* < 0.001).

Within each river, upstream stations showed significantly lower concentrations of proteins and carbohydrates than in the corresponding downstream stations (permutational Welch *t* test, *p* < 0.05 for all stations) except for the RMO (t = 0.83456, *p* value = 0.432 for carbohydrates and t = 1.9518, *p* value = 0.078 for proteins) and RGA (t = − 2.3655, *p* value = 0.094 for proteins) rivers. In downstream sites, when EPS concentrations were significantly different between sampling times within each station (such as the GRG, RMO and RPE stations; one-way ANOVA, *p* < 0.01 for all), carbohydrate concentrations were always significantly higher at T14 than at T07 and T21.

### The friction coefficient showed differences with CLD concentration

**Figure 4:**
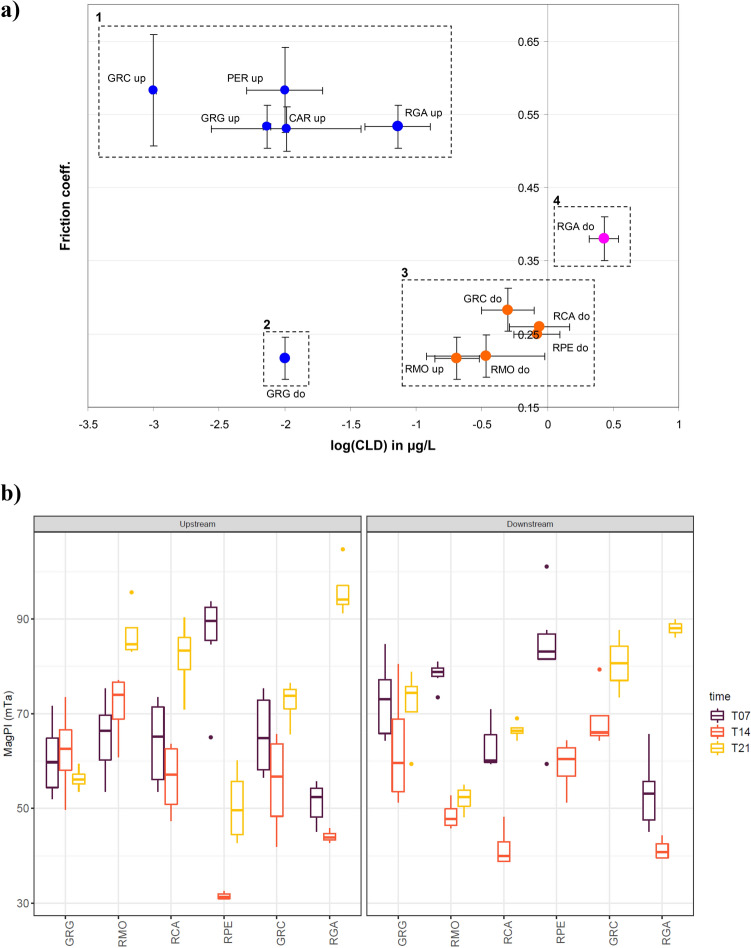
(**a**) The friction coefficient related to the CLD contamination, the same color codes as Fig. 1b are employed, identifying the three contexts of pollution, (**b**) Surface adhesiveness of upstream (left panel) and downstream (right panel) epilithic biofilms measured by Magnetic Particle Induction (MagPI). Sampling sites were ordered from the least polluted to the most polluted according to the order given in Fig. 1b for downstream sites.

Friction coefficients revealed clear differences between biofilms growing in contaminated areas where friction was minimal, and in non-contaminated stations, where friction was at its highest (Fig. 4a). For reference, 0.57 (± 0.029) is the friction coefficient of a non-immersed rock. Four groups were therefore defined: #1 is a group of unpolluted stations, except for the Grande Goyaves River downstream from the banana plantations (GRGdo, #2), while groups #3 and #4 include polluted stations, #4 containing the heavily polluted station. The RMOup station friction coefficient, polluted by CLD *in fine* (see Table 1), places it in group #3 for polluted stations. The GRGdo station, unpolluted but submerged by leaf litter during the experiment, showed a low friction coefficient.

### Surface adhesiveness showed no significant difference between polluted and non-polluted sites

Both in upstream and downstream sites, MagPI measurements (Fig. 4b) revealed variations in epilithic biofilm adhesiveness according to the date and the station but there were no significant differences in biofilm adhesiveness between upstream and downstream sites (three-way ANOVA: time effect F = 79.55, df = 2, *p* < 0.001; station effect F = 5.74, df = 5, *p* < 0.001; site effect F = 1.35, *p* = 0.25; all interactions *p* < 0.01 or 0.001). In downstream sites where MagPI varied significantly between sampling times within each station (e.g., RGA, RCA, GRC, RPE and RMO, one-way ANOVA, *p* < 0.01 for all), biofilm adhesiveness was always significantly lower at T14 than at T07 and T21.

### Viscoelasticity identified three layers in the biofilm

**Figure 5:**
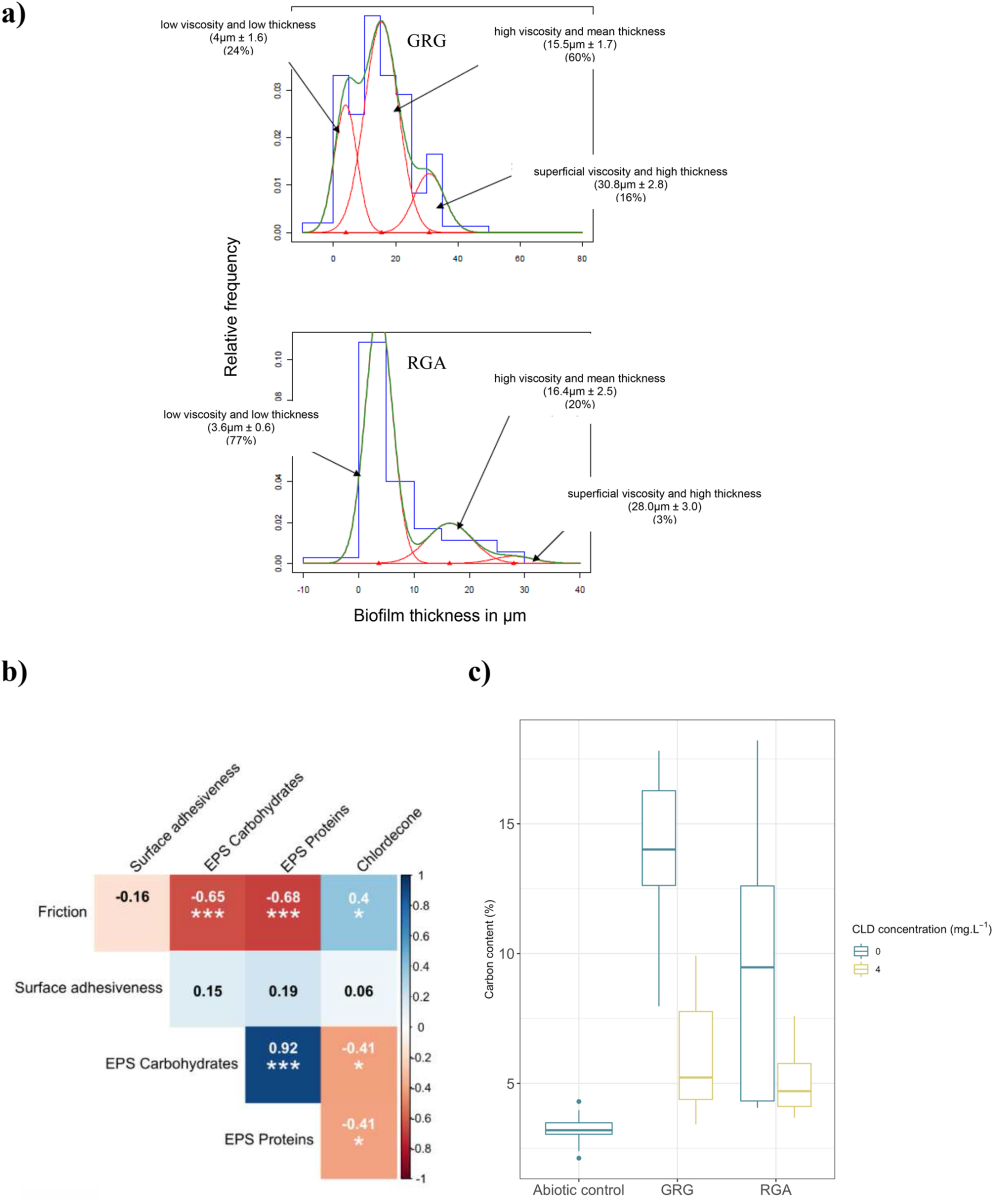
(**a**) biofilm grown in a contaminated (GRG), or non-contaminated (RGA) environments during 21 days, combination of viscoelasticity information upon thickness (histogram) results, (**b**) Correlogram of the main physico-chemical parameters of epilithic biofilms, (**c**) Carbon content (in percent) detected by Energy-Dispersive X-ray spectroscopy (EDXs) analysis applied on samples obtained from the microcosm experiment.

A Young’s modulus that immediately reaches a value near 70 GPa with a phase angle close to zero degrees—which corresponds to the mechanical parameters of a glass substrate—indicates the absence of biofilm in this area. In contrast, modulus and phase angles that vary significantly in terms of indentation depths and from one curve to the next indicate that the indentations were performed on biofilm matter. For reference, typical test curves are shown in supp. mat. S1. At 21 days, measurements revealed a biofilm optimal for indentation analyses (Fig. 5a). In the non-polluted station (GRGdo), 60% of the measurements provided modulus values near 70 GPa with a phase angle of 0 degrees. The remaining 40% measurements qualified a matter for which Young’s modulus values ranged between 0 and 15 GPa (very low values indicative of high elasticity) with a phase angle exceeding 50 degrees (indicative of high viscosity): these values matched those of the biofilm. In the highly polluted station (RGAdo) at T21, 20.5% of the measurements showed a Young’s modulus less than 15 GPa and 79.5% of the indentations were touching the glass. The Fisher and Cripps rule was used to estimate the biofilm’s thickness. Three biofilm layers were identified at the surface of the substrate: a first layer, with a mean thickness of 4 µm and a low viscosity, was very dominant on substrates immersed in the polluted river (RGAdo); a second layer with a mean value of 15–16 µm and a high viscoelasticity was very abundant in the non-polluted station and much less developed in the polluted station; a third thicker layer, with a mean depth of 28–31 µm, was a minority in both environments.

### Correlating biofilm descriptors and CLD pollution

We investigated cross-correlations between the chemical and physical descriptors of the biofilm and the CLD, and their respective levels of significance (Fig. 5b). Within the physical measures, only friction properties showed a significant correlation with CLD levels (r = 0.40, *p* < 0.05), the surface adhesiveness being nonsignificant (r = 0.06, *p* > 0.05). These two ways of measuring the physical properties of the biofilm were not correlated (r = -0.16, *p* > 0.05). The chemical analyses of bacterial EPS showed a strong intercorrelation between carbohydrates and protein fractions, each being positively correlated with the CLD level (r = -0.41, *p* < 0.05 for carbohydrates, r = -0.41, *p* < 0.05 for proteins). The carbohydrates and proteins in the EPS matrix were highly correlated with friction properties with r = -0.65 (*p* < 0.001) and -0.68 (*p* < 0.001) respectively.

### Carbon content in the microcosm experiment impacted by CLD

All slides colonised with biofilm and incubated in the microcosms for 14 days showed a higher carbon content than the abiotic control, exemplifying biofilm growth in microcosms (Fig. 5c). In contaminated conditions (4 µg/L of CDL), the increase was moderate and of the same order of magnitude for both rivers (mean increase of 2%). In contrast, carbon content indicated a better growth (from 6 to 11%) in non-contaminated conditions; the highest increase was observed in the non-polluted river (i.e., GRG). Using analysis of variance, the difference in carbon content between rivers was not significant (F = 2.3463, *p* = 0.1495), whereas the difference between treatments was strongly significant (F = 14.4077, *p* = 0.0022) and interaction was not significant (F = 0.8754, *p* = 0.3679), the differences between shelf position (*p* = 0.6128) and placement on the shelves (*p* = 0.8546) being not significant.

## Discussion

The study we present took place in the unusual context of small islands of the French West Indies. In consequence small distances (2.6 to 7.5 km) between "upstream" and "downstream" stations, consequence of the spatial constraint on the island (Fig. 1a), introduced strong homogeneity between pairs of stations. The sampling sites were therefore situated in water blades of similar temperatures, oxygenation, pH ranges (Fig. 1b), located in identical calm facies (*sensus* Malavoi ^47^) and differed mainly by agronomic situations conducted on their riverbanks. A difference in the total mineral load observed for the RCAR and RGA rivers justified a pair-wise reasoning between upstream and downstream sites to ensure maximum similarity between sites, excluding the pollutant. To place CLD contamination values obtained in this study in a broader context, we’ve summarised the 2069 pesticides analyses of the mandatory State environmental surveys obtained at the same samplings sites and during the same period (i.e., between May 2012 and May 2013) (supplementary material S2). No heavy metal or organic pollution was diagnosed in the rivers. These results reveal the fugitive presence of 7 out of a total of 247 compounds among which four belong to the PAH group and one compound which shows multiple biogenic origins: indeno (1,2,3-cd) pyrene which occurs naturally in the ground and in vegetation. The most significant and regularly found compound is confirmed to be CLD, which can accumulate in the food chain in concentrations as low as 0.023 µg/L^48^ and is shown to have a strong effect even at low concentrations. These data confirm the wide prevalence of the CLD molecule in the rivers sampled and its strong putative effects on the aquatic environment.

Considering the effects of this pollution on the components of the epilithic biofilm, diatoms were intensively studied for purposes of ecological evaluation of the pollution levels due to their excellent ability to serve as indicators for nutrients and organic matter pollution^49–51^. Direct effects of insecticides or acaricides on diatom species have been demonstrated by recent studies showing their altered photosynthetic capacity in presence of pyrethroids^52^ and a reduced cell count in presence of organochlorinated pesticides^53^ or organophosphates^54^. An indirect effect of insecticides on diatom assemblages has also been demonstrated through the inhibition of bacterial signaling molecules in studies conducted on biofouling mechanisms^55^. Quorum-sensing signals originating specifically in gram-negative bacteria were involved in the alteration of the diatom-biofilm relationship, accompanied with changes in chlorophyll *a* and EPS content^56^. When seeking variations in diatomic taxonomic assemblages in situ, our results have shown that the heterogeneity of communities is such that the data is not suitable to serve as a contamination indicator. The length of time and the difficulties necessary to acquire taxonomic information from tropical countries actually favours other metrics, such as life forms, cell sizes (or biovolumes) and ecological guilds which are all possibilities for purposes of ecological studies and bioassessments^57–59^. We thus prospect for alternative indicators. Diatom motility has been directly linked to water pollution^58,60^ and this guild is described as more tolerant, with a proposed hypothesis of a protective effect of the EPS matrix^61^. The 3D structure of the first layer of the biofilm during its construction has been demonstrated as a result of the ability of primo-colonizing microbes to elaborate self-produced matrix modifying complex interstitial flow velocities, concavities and solutes repartition^28^. It has been also shown that under adverse environmental conditions, bacteria secrete EPS rather than proliferate in order to provide mechanical stability to their surroundings^27^. Our results are consistent with these studies, which show that taxonomic information is not indicative of the level of pollution but that the prevalence of motile diatoms in contaminated rivers was a more reliable indicator that suggests an indirect effect of EPS production during bacterial primocolonization on subsequent diatom installation.

We also use chemical element mapping via Energy Dispersive X-ray analysis (EDX) technique to investigate biofilm response to CLD contamination. This technique was employed to monitor changes in cell wall chemical composition in biofilm cultures^62^, loss of biomass and biodiversity in biofilms polluted by nanoparticles^63^, micro-structural characterisation of exolithobiontic microbial biofilms^64^, or biofilm colonisation quantification on porous composite membranes^65^. In the present study, the carbon content measured on glass slides incubated in the microcosm experiment clearly indicated that CLD altered the epilithic biofilms irrespective of its origin. Importantly, this result indicates that CLD disrupted RGA biofilm development and growth even though the native microorganisms are recurrently exposed to CLD in their natural context. This indicates that, when looking at a structural descriptor such as growth, an RGA community faced with a high, albeit realistic, concentration of CLD, has not better adapted than the less exposed biofilm community. The effects of CLD on the survival and growth of freshwater microorganisms—demonstrated since the 1980s^15,16^ pointed out a strong decrease in aerobic bacterial growth caused by the undermining of their oxidative functions^17^. This pioneering work showed that NADH oxidase and succino-oxidase activity was depleted by 51% and 25% respectively upon the addition of 1 µM of CLD to the bacterial cultures, and a modulation of the NADH oxidase gene has also been demonstrated as reducing bacterial growth rate with a higher carbon source being saved to be used for EPS biosynthesis^66^. In our study, the analyses of the EPS matrix showed significant differences in carbohydrate and protein concentrations, with upstream stations having significantly lower concentrations than in the corresponding downstream contaminated stations. In addition and as evidenced by the decrease in carbon content on CLD impacted biofilms, we may hypothesise that protein and carbohydrate secretions were also affected in terms of composition.

The EPS matrix controls the cell surface charge and hydrophobicity in biofilms and deoxy sugars such as fucose, rhamnose, and pentose are known to enhance the hydrophobicity of biofilms^67^. Within the physical properties of biofilms, the friction coefficient is highly sensitive to the hydrophobic nature of the test substratum and it is significantly higher on hydrophobized silica than on a flat reference hydrophilic silica^68^. Any changes in the hydrophobic nature of epilithic biofilms (likely induced by the modification of the extracellular metabolite pool) is thereby expected to have a significant impact on friction properties. Our results (Figs. 4a, 5b) showed that the friction is globally minimized in contaminated situations and our results have pointed out a mesoscale chemical and physical reactivity of the biofilm that can be correlated with CLD contamination in polluted and unpolluted stations. Not all physical variables tested were reactive to CLD due to a lack of significant MagPI measures (adhesiveness) in this case, whereas friction properties have proven to show a very good correlation with CLD concentration as well as EPS carbohydrates and proteins concentrations.

**Figure 6:**
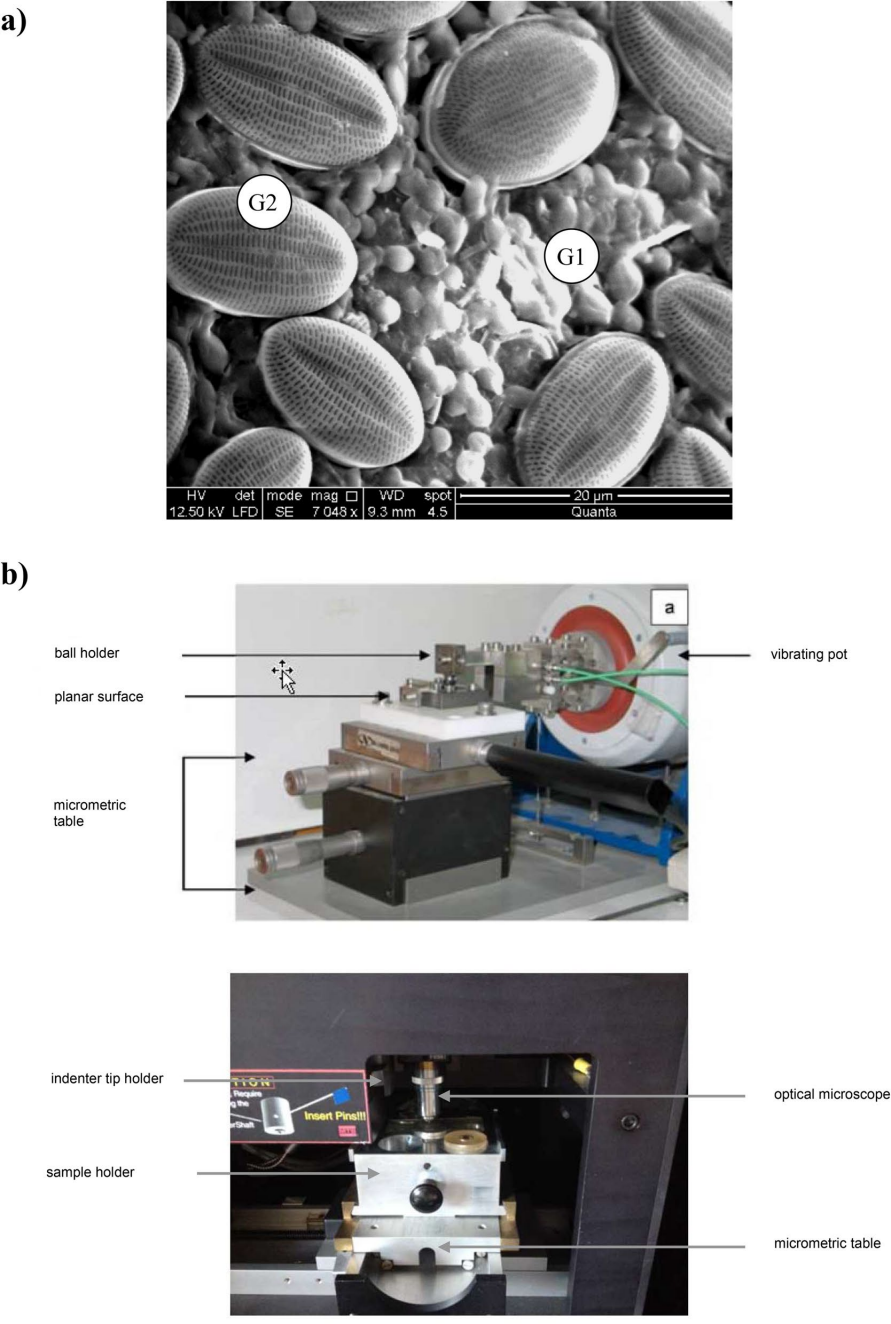
(**a**) Image showing the two first superimposed layers (bacterial G1 and bacteria + small diatoms in G2) during colonization process, (**b**) experimental alternative tribometer sphere/plane under ambient atmosphere (upper panel, conception and realisation GTSI team), and XP nanoindenter (lower panel). The images in this figure were taken by the authors.

Less practicable for routine testing, the microindentation technique used to infer the visco-elastic properties of the biofilm has proven in our study to be a useful tool to build hypotheses on the mechanisms underlying CLD impact during the primary phase of colonisation, mechanisms responsible of the demonstrated changes in its EPS composition and growth. Our results identified three layers as structurally characteristic of early biofilm: (1) a group defined by a 4 µm thick layer (G1) which could be suggested as consisting of bacteria embedded in a thin layer of EPS, (2) a group with a mean thickness of 16 µm (G2) which could be suggested as containing the first stages of other organism colonisations (Fig. 6a), and (3) the group containing big diatoms and/or more complex or mature EPS network (G3), with high superficial viscosity. When studying the proportion variations of these groups in biofilm growing in contaminated and non-contaminated stations, the G1 → G2 → G3 succession appears to have been mitigated in the contaminated area (with G1 representing 77% in the polluted station versus 24% in the non-polluted station), tempting to suggest a delay in the construction of the epilithic biofilm in contaminated situation. This data confirms the often observed “vitrified aspect” of epilithic biofilm in CLD-contaminated waters and the differences observed in EPS and friction properties in our study.

## Conclusion

The epilithic biofilm is the main indigenous primary producer in Caribbean rivers. In this context, the reduction of biofilm biomass due to CLD pollution, as attested in the microcosms experiment, is of concern when transposed at the ecosystem level because it would limit resource availability to higher consumers. Our results have also shown modifications in the secretion of extracellular carbohydrates and proteins due to pollution that could modulate the energy support provided by the consumption of biofilm, especially among juveniles of aquatic species, large consumers of this source of organic carbon^69^ which is easier to assimilate. This work has also revealed interesting relationships between the physical characteristics of epilithic biofilms and the exopolymer secretions in relation to CLD pollution. These results give rise to a new area of research in the fields of bioindication and freshwater environmental assessment in rivers polluted by pesticides. Lubrication is particularly promising in this context as this is straightforward, a time-saving measure in comparison to usual diatoms inventories.

## Methods

### Sampling

Two different approaches were used in this study, one in situ in the rivers of the Basse-Terre region of Guadeloupe, whereas the second used microcosms that were set up in the laboratory. Two sampling campaigns were conducted. For the first campaign, six rivers were chosen and sampled in February 2013 (Supplementary material S2b) on the basis of their content in CLD and their ability to both contain pristine waters upstream from the banana plantations and contaminated waters downstream from the banana fields. The stations were chosen to cover a range of situations between the lowest levels of CLD in the Grande Goyaves River (GRG), to the highest levels in the Grande Anse River (RGA)^70^. The usual parameters collected from national surveys are provided in Fig. 1a and Supp. mat. S2d. For each of the twelve stations, water samples (n = 4) were collected at T0 day (time of immersion), T7 (7 days later), T14 (14 days later) and T21 (21 days later) in glass bottles sterilised beforehand at 450 °C. Samples were kept with freeze packs during transportation and stored at -20 °C until analysis. At T21, the mature epilithic biofilm was scraped from the surface of submerged rocks in the river bed with a knife and a brush according to the national standard (20 cm under the surface and at least 100 cm^2^ collected on at least five stones). Each sample was fixed in formaldehyde at a final concentration of 10%. A series of andesitic rocks collected in the river and cut into 35 cm^2^ slices were used as artificial substrates for immersion and investigation by means of tribological analyses.. These were located in the same calm hydro-morphodynamic facies (pools) in order to avoid the effects of a putative blurring factor such as turbulence. Ten replicates of microscope glass slides were also immersed in baskets specially designed, some of which were collected at T7, T14 and T21 (Supp. mat. S2c).

The second sampling campaign was undertaken in April 2015 in stations where the two extremes of the river CLD contamination described above had been observed, i.e., downstream of the banana plantations in the Grande Goyaves River (GRG) and downstream of the banana plantations in the Grande Anse River (RGA). The objectives aimed to reinforce previously observed results by taking advantage of a microcosm experiment where only the level of CLD varied while other physicochemical parameters^71^ were being controlled. Batches of 10 microscope glass slides were immersed for 28 days, the glass slides for the microcosm experiment were pulled out of the water at 10 days while those for the microindentation analyses were removed at T14, T21 and T28. The slides were transported immersed in the water of their respective river station and kept at 5 °C to avoid any degradation and processed the day of collection.

### Samples processing

#### Chlordecone concentration

The samples were thawed before extraction and processed without a filtration step because of the low quantity of suspended matter present in the samples. Water samples of 100 mL were spiked with an internal standard solution (chlordecone C13) and were extracted three times with 10 mL of dichloromethane. The final extract was transferred into 100 µL of acetonitrile for injection. The extracts were analysed by HPLC–ESI–MS-MS (UPLC-Quattro premier Waters). Separation was achieved using a BEH-C18 column (50 mm x 2.1 mm; 1.7 µm) at a temperature of 35 °C. Acetonitrile and water (ultrapure deionised water) were used as the mobile phases. Quantification was performed in accordance to internal standards. The analytical method was validated in terms of calibration linearity, specificity, extraction recoveries (93 ± 8%), and limits of quantification (2 pg injected or 1 ng/L). All solvents for the chemical analyses, dichloromethane (DCM) and acetonitrile (ACN) (HPLC reagent grade, Scharlau) were purchased from ICS (Belin Beliet, France). Analytical standards of chlordecone were purchased from LGC Standards (Molsheim, France). The analytic methodology consisted of a liquid/liquid extraction (LLE) followed by an analysis in liquid-phase chromatography (LC) coupled with a mass spectrometry in tandem (MS/MS). The analytical performances obtained in terms of limit of quantification and reproducibility were LQ from 1 to 5 ng/L and variability lower than 15%. For details on the techniques, see^72,73^.

#### Exopolymeric substances (EPS)

Colloidal and bound EPS from the biofilms were extracted by rotating the glass slides in a mixture of distilled water and ion-exchange resin (Dowex Marathon C, sodium, Sigma) for 1.5 h at 4 °C. Practically, glass slides were placed in 50 mL tubes filled with the mixture in order to be immersed during the agitation procedure. The exopolymer solution was then retrieved and freeze-dried. Carbohydrate and protein analyses were performed following the phenol assay protocol^74^ and the modified Lowry procedure^75^, respectively. For colloidal carbohydrate analyses, 200 μl phenol (5%) and 1 mL sulphuric acid (98%) were added to 200 μl of subsamples. They were then incubated for 35 min at 30 °C and the carbohydrate concentration was measured with a spectrophotometer at 488 nm (Milton Roy Spectronic Genesys 2). For colloidal protein analyses, 250 μl subsamples were incubated for 15 min at 30 °C with 250 μl of 2% sodium dodecyl sulphate salt (SDS) and 700 μl of a chemical cocktail prepared as described in Dubois et al. (1956)^75^. The subsamples were then incubated for another 45 min at 30 °C with 100 μl of Folin reagent (diluted with distilled water 5:6 v/v). The protein concentration was measured by spectrophotometry at 750 nm. Calibration curves were prepared using glucose and bovine serum albumin (BSA) as standards for carbohydrates and proteins, respectively.

#### Diatoms

Samples were treated with hot concentrated hydrogen peroxide (H_2_O_2_ 30%) to eliminate protoplasms and, when appropriate, with hydrochloric acid (removal of carbonates). After drying, the diatoms were mounted in refractive resin, Naphrax (Northern Biological Supplies Ltd., England—Refractive index = 1.74) and identified through microscopic examination. The counting protocol and identification of diatom valves is defined by the European Standard EN 14407 2004 and Stoermer et al*.* (1996)^76^. Four hundred diatom valves were counted and identified to the species level for each sample.

#### Tribological analysis

The analyses were performed with a tribometer using an alternative ball-on-plane tribometer consisting of an AISI 52100 steel ball rubbing against an AISI 52100 steel plane, on which the tested material is deposited (Fig. 6b).

#### Magnetic particle induction

Ferrous particles (diameter > 425 μm) were added in a single layer to the biofilm surface and subjected to an increasing magnetic field produced by an incremental increase in the current supplied to the electromagnet. The electromagnet was set 3 mm away from sediment surface and connected to a variable voltage power supply (HY3005 DC Power Supply, Mastech). Voltage was increased from 0 V by increments of 0.1 V until all particles detached from the sediment. The voltage (i.e., strongly correlated to the magnetic force) at which the particles are recaptured is determined as a measure of surface adhesion. This final voltage was recorded and the associated magnetic force was determined using a calibration curve previously established with a gaussmeter (410-HCAT, Lake- Shore). This magnetic flux (mT) was used as a measure of surface adhesiveness.

#### Nanoindentation analyses

The measurements were achieved with a nanoindentor XP from MTS (Agilent Corp.) equipped with a CSM module (Continuous Stiffness Measurement) (Fig. 6c). In CSM mode, a sinusoidal force is superimposed to the quasi-static loading allowing for the measurement of mechanical parameters as a function of the indentation depth. The phase angle represents the phase shift between force and displacement during a dynamic test and is essential to reveal the viscosity properties of a material. The quasi-static load was applied with a strain rate of 0.05 s^−1^ and the tests were carried out using a Berkovicth diamond tip. The glass slides were pulled out of the water at T14, T21 and T28. The Young’s modulus and hardness were deduced from the load–displacement curves using the Oliver and Pharr method^77^. Seventy-five indents were randomly performed on each slide in order to study the homogeneity of the film grown on the glass substrates. The thickness of the biofilm was estimated by Fisher and Cripps’s “rule of thumb” ^78^.

#### Microcosm experiment

Two treatments were considered (control and chlordecone) for each river (GRG and RGA) along with one abiotic control that contained non-colonised slides. Each condition was performed in quadruplicates (four independent microcosms) in a phytotron (MLR351H Sanyo Electric Co. Ltd. Japan): assays with CLD (n = 4 × 2), biotic controls without CLD (n = 4 × 2), abiotic controls (n = 4). Once in the laboratory, two slides were immediately stored at -20 °C while the other slides were transferred to rectangular sterile glass containers (around 1 L volume) filled with 920 mL of exposure solutions. Exposure solutions were prepared with pesticide-free water collected from the Pérou River (Capesterre, Guadeloupe), the reference of a pristine river for studies conducted on the subject of water contamination by chlordecone in Guadeloupe^79^. The water from this river was filtered through 0.22 µm cellulose nitrate filters (Sartorius Goettingen, Germany) in order to exclude the input of foreign microorganisms from the experiment. Spiked microcosms were prepared with the realistic environmental nominal chlordecone concentration of 4 µg/L, in accordance with the highest concentrations encountered in the RGA river, whereas the control did not contain additional chlordecone but only the same quantity of methanol used to prepare the chlordecone solution. The abiotic control was set up with a solution spiked with chlordecone at 4 µg/L. Every container was randomly placed in a growth chamber MLR351H (Sanyo Electric Co. Ltd. Japan). The photoperiod was set to follow local tropical day/night rhythms with respect to natural river shaded environments (light programmed from 0 to 5000 lx depending on the hour), and temperature was set at 25 °C. The 5000 lx represents 10% of the maximum incident illumination measured on an uncanopied epilithic biofilm in a tropical river (95 μmol/m^2^/s versus 1000 μmol/m^2^/s^80^) and was chosen to avoid over-lighting the samples originating from canopied environments. All microcosms were aerated with aquarium pumps through rubber pipes fitted with 0.22 µm polyethersulfone (PES) syringe driven filters (Millex Carrigtwohill, Ireland) and a 1 mL polypropylene sterile syringe (Terumo, Leuven, Belgium). Containers were covered with plastic wrap to avoid CLD concentration due to evaporation, as well as to preclude contamination across containers. The water level was checked over the duration of the experiment by using a gauge line as a point of reference. Slides were collected at 14 days and immediately stored at -20 °C.

#### Energy-dispersive X-ray spectroscopy (EDXs) analysis

Taking into account the elemental composition of the glass support (exempt of C), the carbon was used as a “proxy” of the biofilm colonisation. In order to detect carbon on the surface of the glass slides colonised by biofilm, frozen slides collected in the microcosms were thawed, kept hydrated and quickly placed in an Environmental Scanning Electronic Microscope (FEI Quanta 250) operating at 15 kV under an environmental pressure of 70 Pa (Low vacuum mode). Energy-dispersive X-ray (EDX) measurements were conducted with an M-max 50 mm^2^ Oxford detector. Elemental mappings were obtained on uncoated samples after 400 s of acquisition time through each slide observed from 4 different areas randomly selected.

#### Statistical analyses

These were performed in an R statistical framework (R version 3.2.3 for Windows) and the RStudio interface version 0.99.486. Hierarchical clustering was used to determine similarities between diatom assemblages^81^ using the “stats” package and the Ward method^82^ on squared Euclidean distances. The Mean Split Silhouette (MSS) criterion^83^ was used to evaluate clustering relevance. Analyses of microindentation data were performed using the “mixdist” package. The analysis of variance (ANOVA) was used to test differences between sampling sites and time for EPS proteins and carbohydrates as well as for surface adhesiveness. Homoscedasticity was tested using Levene’s Test of Equality of Variances (“lawstat” package) and normality was tested using Shapiro–Wilk Normality Test on model residuals. Simple Correlation was calculated between the physicochemical descriptors of the biofilm using the Pearson coefficient and the correlogram was designed using the “corrplot” package.

### Ethical approval

This article does not contain any studies with human participants or animals performed by any of the authors.

## Supplementary information

Supplementary Information.

## Data Availability

All data needed to evaluate the conclusions in the paper are present in the paper and/or the supplementary materials. Material related to this paper may be requested from Dominique Monti (dominique.monti@univ-antilles.fr), Cédric Hubas (cedric.hubas@mnhn.fr) and Béatrice Lauga (beatrice.lauga@univ-pau.fr).
