# Supplementary material for: Physical properties of epilithic river biofilm as a new lead to perform pollution bioassessments in overseas territories
Source: Sci Rep. 2020 Oct 14;10:17309. doi: 10.1038/s41598-020-73948-7 (PMC7560750; doi:10.1038/s41598-020-73948-7)
Supplement: Supplementary file 1 — Supplementary Information. [file 41598_2020_73948_MOESM1_ESM.pdf]

Physical Properties of Epilithic River Biofilm As a New Lead to Perform Pollution Bioassessments in Overseas Territories

Dominique Monti[1,9], Cedric Hubas[2], Xavier Lourenço[1], Farid Begarin[4], Alexandre Haouisée[1], Laurence Romana[5], Estelle Lefrançois [6], Alexandra Jestin[7], Hélène Budzinski[8], Nathalie Tapie[8], Théo Risser[10], Jean-Louis Mansot [4,5], Philippe Keith[3], Olivier Gros[4,9], Pascal-Jean Lopez[3] and Béatrice Lauga[10]

Supplementary material S1

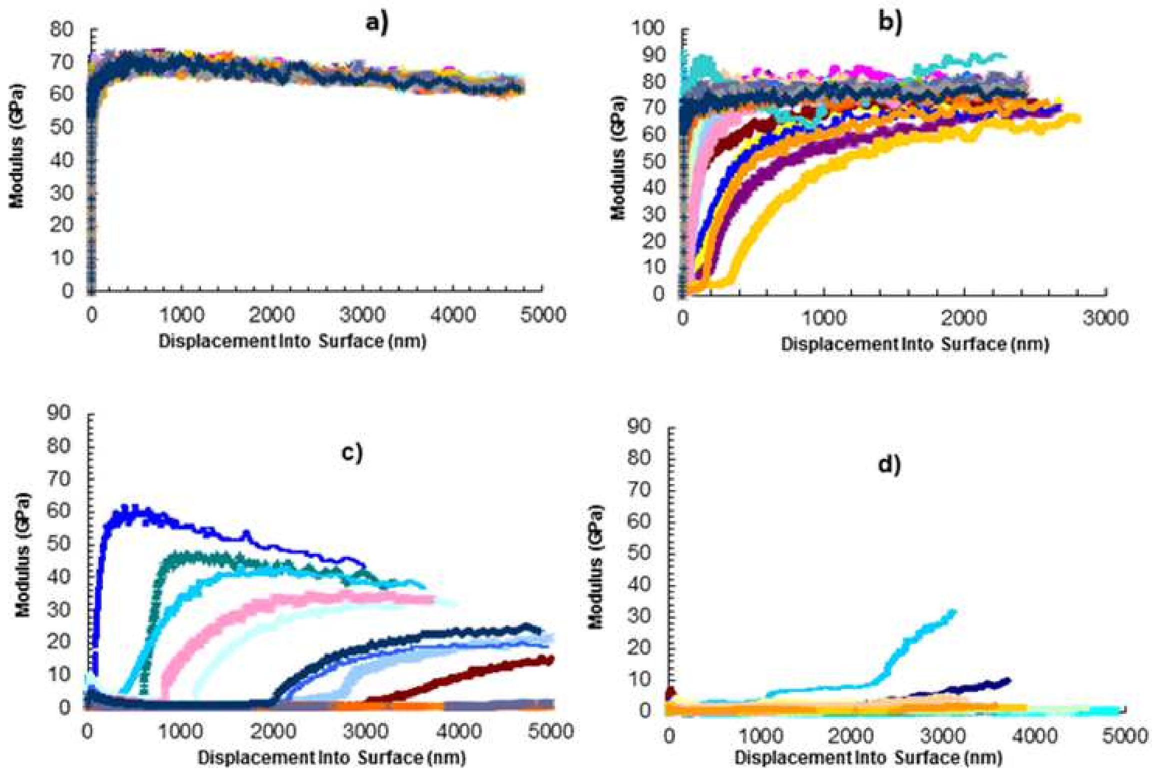

Supp. mat. S1: Typical curves showing the Young's modulus and phase angle as a function of indentation depth. a) viscoelastic properties of glass and properties of biofilm visible at b) T14, c) T21, d) T28.

Supplementary material S2

a)

River	Compound	Concentration (µg/L)
RCA	Benzo(a)pyrene	0.001
RCA	Benzo(a)pyrene	0.001
GRC	Benzo(a)pyrene	33
GRC	Benzo(a)pyrene	0.001
GRC	Benzo(a)pyrene	0.001
GRG	Benzo(a)pyrene	0.001
GRG	Benzo(a)pyrene	0.001
RMO	Benzo(a)pyrene	0.001
RMO	Benzo(a)pyrene	0.001
RCA	Benzo(b)fluoranthene	0.005
RCA	Benzo(b)fluoranthene	0.005
GRC	Benzo(b)fluoranthene	38
GRC	Benzo(b)fluoranthene	0.005
GRC	Benzo(b)fluoranthene	0.005
GRG	Benzo(b)fluoranthene	0.005
GRG	Benzo(b)fluoranthene	0.005
RMO	Benzo(b)fluoranthene	0.005
RMO	Benzo(b)fluoranthene	0.005
RCA	Benzo(ghi)perylene	0.005
RCA	Benzo(ghi)perylene	0.005
GRC	Benzo(ghi)perylene	32
GRC	Benzo(ghi)perylene	0.005
GRC	Benzo(ghi)perylene	0.005
GRG	Benzo(ghi)perylene	0.005
GRG	Benzo(ghi)perylene	0.005
RMO	Benzo(ghi)perylene	0.005
RMO	Benzo(ghi)perylene	0.005
GRC	Benzo(k)fluoranthene	17
RCA	Benzo(k)fluoranthene	0.005
RCA	Benzo(k)fluoranthene	0.005
GRC	Benzo(k)fluoranthene	0.005
GRC	Benzo(k)fluoranthene	0.005
GRG	Benzo(k)fluoranthene	0.005
GRG	Benzo(k)fluoranthene	0.005
RMO	Benzo(k)fluoranthene	0.005
RMO	Benzo(k)fluoranthene	0.005
RCA	Chlordecone	1.22
RCA	Chlordecone	2.8
GRC	Chlordecone	0.85
GRC	Chlordecone	1
RGA	Chlordecone	2.6
RMO	Chlordecone	0.32
RMO	Chlordecone	0.15
RCA	HCH Beta	0.16
RCA	HCH Beta	0.12
RCA	HCH Beta	0.02
RCA	HCH Beta	0.04
GRC	HCH Beta	0.02
GRC	HCH Beta	0.02
GRC	HCH Beta	0.01

River	Compound	Concentration (µg/L)
GRC	HCH Beta	0.01
RGA	HCH Beta	0.20
GRG	HCH Beta	0.01
GRG	HCH Beta	0.01
RMO	HCH Beta	0.09
RMO	HCH Beta	0.13
RMO	HCH Beta	0.06
RMO	HCH Beta	0.07
GRC	Indéno(1,2,3-cd)pyrène	24
RCA	Indéno(1,2,3-cd)pyrène	0.03
RCA	Indéno(1,2,3-cd)pyrène	0.03
GRC	Indéno(1,2,3-cd)pyrène	0.03
GRC	Indéno(1,2,3-cd)pyrène	0.03
GRG	Indéno(1,2,3-cd)pyrène	0.03
GRG	Indéno(1,2,3-cd)pyrène	0.03
RMO	Indéno(1,2,3-cd)pyrène	0.03
RMO	Indéno(1,2,3-cd)pyrène	0.03

c)

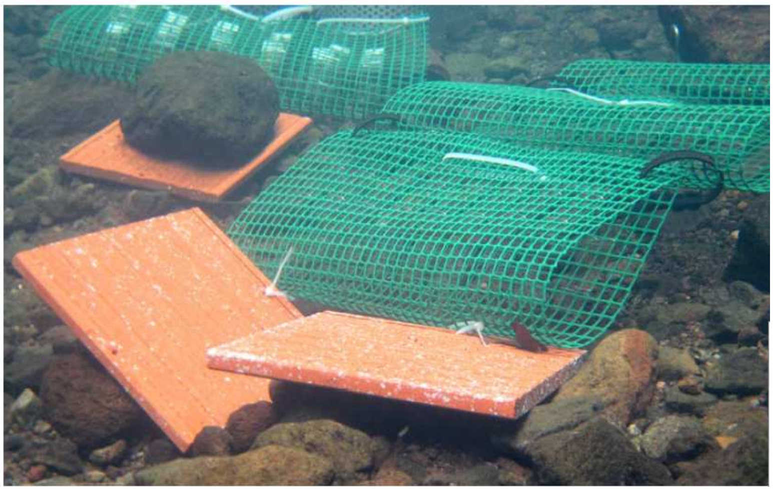

b)

			February 2013									April 2015				
river	local.	abbrev.	CLD in water				Diatoms	EPS			Friction	CLD			Microcosm	Microindent.
			T0	T7	T14	T21	T21	T7	T14	T21	T21	T0	T14	T21	T14	T21
Capesterre	downstr. ban.	GRCdo	X	X	X	X	X	X	X	X	X					
Carbet	downstr. ban.	RCAdo	X	X	X	X	X	X	X	X	X					
Grande Anse	downstr. ban.	RGAdo	X	X	X	X	X	X	X	X	X	X	X	X	X	X
Gde Goyaves	downstr. ban.	GRGdo	X	X	X	X	X	X	X	X	X	X	X	X	X	X
Moustique	downstr. ban.	RMOdo	X	X	X	X	X	X	X	X	X					
Pérou	downstr. ban.	RPEdo	X	X	X	X	X	X	X	X	X					
Capesterre	ahead ban.	GRCup	X	X	X	X	X	X	X	X	X					
Carbet	ahead ban.	RCAup	X	X	X	X	X	X	X	X	X					
Grande Anse	ahead ban.	RGAup	X	X	X	X	X	X	X	X	X					
Gde Goyaves	ahead ban.	GRGup	X	X	X	X	X	X	X	X	X					
Moustique	ahead ban.	RMOup	X	X	X	X	X	X	X	X	X					
Pérou	ahead ban.	RPEup	X	X	X	X	X	X	X	X	X					

d)

	GRG	RMO	GRC-RPE	RCA	RGA
Turbidity FNU	0.949	1.16	0.471	0.367	0.68
Suspended matter (Whatman Grade GF/A filter)	2.2	<2	<2	<2	<2
BOD5	1.1	2.5	2.4	2.6	6.9
Chemical Oxygen Demand	<30	<30	<30	<30	<30
Total Kjeldahl nitrogen (in N)	<0.4	<1	1.46	<1	<1
Ammonium	<0,05	<0,05	<0,05	<0,05	<0,05
Nitrites	<0,05	<0,05	<0,05	<0,05	<0,05
Nitrates	<1	<1	1.704	1.626	<1
Dissolved silica (in mg/L of SiO2)	20.1	17.13	10.65	22.16	24.64
Total phosphorus (in P2O5)	<0.11	<0.11	<0.11	<0.11	<0.11
Potassium	<1.00	<1.00	<1.00	1.85	<1.00
Magnesium	1.83	1.56	1.65	4.33	1.1
Calcium	5.09	4.8	5.31	13.5	4.02
Sodium	6.39	4.94	4.5	8.1	3.94
Orthophosphates (in PO4)	<0,1	<0,1	<0,1	<0,1	<0,1
Phaeopigments	2.5	<2.0	<2.0	<2.0	<2.0
Alfa chlorophyll	<0.5	<0.5	<0.5	<0.5	0.7
Dissolved organic carbon (DOC)	1.39	1.32	1.97	1.23	0.715

Supp. mat. S2: a) Pesticides detected at least one time during the year before and including our sampling campaign (four campaigns between January 2012 and March 2013, mandatory National Surveys[72]), b) Times of data collection, c) Underwater view of microscope slides, d) Chemical analyses realized in the downstream stations in February-March 2013 (mandatory National Surveys[72], GRC and RPE being sampled just below their confluence).
